# Design, synthesis and biological evaluation of selective survivin inhibitors

**DOI:** 10.7555/JBR.31.20160173

**Published:** 2019

**Authors:** Min Xiao, Yi Xue, Zhongzhi Wu, Zi-Ning Lei, Jin Wang, Zhe-Sheng Chen, Wei Li

**Affiliations:** 1. Department of Pharmaceutical Sciences, University of Tennessee Health Science Center, Memphis, TN, 38163, USA; 2. Department of Pharmaceutical Sciences, College of Pharmacy and Health Sciences, St. John’s University, Queens, NY 11439, USA.

**Keywords:** selective survivin inhibitors, structure activity relationships, melanoma, human epidermoid carcinoma, colorectal cancer, P-glycoprotein drug efflux pumps

## Abstract

The differential distribution between cancer cells and normal adult tissues makes survivin a very attractive cancer drug target. We have previously reported a series of novel selective survivin inhibitors with the most potent compound MX106 reaching nanomolar activity in several cancer cell lines. Further optimization of the MX106 scaffold leads to the discovery of more potent and more selective survivin inhibitors. Various structural modifications were synthesized and their anticancer activities were evaluated to determine the structure activity relationships for this MX106 scaffold. *In vitro* anti-proliferative assays using two human melanoma cell lines showed that several new analogs have improved potency compared to MX106. Very interestingly, these new analogs generally showed significantly higher potency against P-glycoprotein overexpressed cells compared with the corresponding parental cells, suggesting that these compounds may strongly sensitize tumors that have high expressions of the P-glycoprotein drug efflux pumps. Western blotting analysis confirmed that the new MX106 analogs maintained their mechanism of actions by selectively suppressing survivin expression level among major inhibitors of apoptotic proteins and induced strong apoptosis in melanoma tumor cells.

## Introduction

Survivin is the smallest (molecular weight 16.5 kDa) member of the IAP (Inhibitor of Apoptosis Protein) family^[1^–^2]^. It is a unique anti-tumor therapy target because: (a) it acts as an essential anti-apoptosis guard which protects cells from the apoptotic cascade by binding to the activated caspases and neutralizing pro-apoptotic receptor^[3^–^5]^; (b) it is a key regulator of mitosis as a component of the chromosomal passenger complex (CPC)^[6^–^7]^; (c) it widely participates in tumorigenesis signaling pathways such as Akt, p53, Wnt-2 and MDM2^[8^–^10]^; (d) it is highly expressed in most types of human cancer cells and embryonic tissues where rapid cell growth is needed, but has very low expression in normal adult differentiated tissues^[11^–^12]^; (e) its expression level is closely correlated with tumor metastasis, poor disease prognosis, and high risk of chemo/radio-resistance^[8^,^11^,^13^–^17]^.

However, due to the nature of survivin function (protein–protein interaction) and its cell cycle dependent expression, the existing armory of survivin inhibitors is still very limited^[8^,^18]^. Most current small molecular survivin inhibitors do not directly bind to the survivin protein, but rather interfere with survivin production (gene transcription, post-translation modification) or accelerate its degradation^[8^,^12^,^18^–^20]^.

Multidrug resistance (MDR) remains a major obstacle in cancer chemotherapy, accounting for more than 90% failure in clinical treatment to cancer patients. A family of ATP binding cassette (ABC) transporters, which is a class of drug efflux pumps, plays a key role in developing MDR^[21]^. At least 15 human ABC transporters, such as *P*-glycoprotein (*P*-gp), multidrug resistant protein1 (MRP1) and breast cancer resistant protein (BCRP) have been found to mediate MDR by inducing drug efflux^[22]^. Among them, *P*-gp has been the most extensively identified MDR in cancer cells and a broad range of chemotherapy drugs are known to be substrates transported by *P*-gp. As the most potent small molecule survivin inhibitor reported so far, YM155 is a survivin gene promotor inhibitor and is well known to be highly susceptible to clinically relevant MDR, including but not limited to *P*-gp overexpres-sion^[18^–^19^,^23^–^24]^. It also shows potential toxicities, which calls for special dose administration arrangements. Therefore, it is important to develop novel selective survivin inhibitors that can overcome clinically relevant MDR.

**Fig.1:**
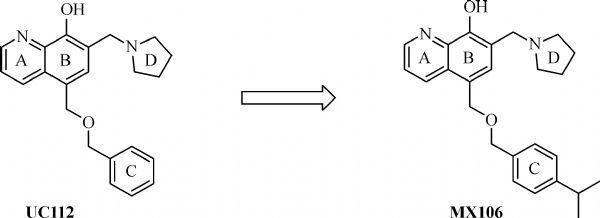
Structures of our previously reported compounds UC112 and MX106

Utilizing high throughput virtual screening and biological test oriented strategy, our laboratory has previously developed a platform of cell permeable small molecular survivin inhibitors, which selectively and effectively reduced survivin expression level in human melanoma and prostate cancer cells, with potent anti-tumor growth efficacy as validated by a human melanoma xenograft model^[25^–^26]^. UC112 is the lead compound we identified through virtual screening and *in vitro* biological studies. Preliminary optimization of UC112 leads to the discovery of a potent and selective survivin inhibitor, MX106. The structure of UC112 and MX106 are shown in ***Fig. 1***. In this report, we described our efforts to further optimize the MX106 scaffolds which lead to the discovery of more potent MX106 analogs for selective survivin inhibition in tumor cells. We report that these new analogs show strong anti-proliferative potency against human melanoma and colorectal cancer cells, effectively overcome *P*-gp mediated drug resistance, and maintain their mechanisms of action by selectively inhibiting survivin expression and inducing cancer cell apoptosis. Structure-activity relationships (SAR) are determined for these new analogs to support further development of this unique scaffold as a potential anticancer agent.

## Materials and methods

### General

All reagents were purchased from Sigma-Aldrich Chemical Co., Alfa Aesar (Ward Hill, MA), and AK Scientific (Mountain View, CA) and were used without further purification. Routine thin layer chromatography (TLC) was performed on aluminum-backed Uniplates (Analtech, Newark, DE). NMR spectra were obtained on a Varian Inova-500 spectrometer (Agilent Technologies, Santa Clara, CA) or a Bruker Ascend 400 (Billerica, MA) spectrometer. Chemical shifts are reported as parts per million (ppm) relative to TMS in CDCl_3_. High resolution mass spectra were collected in positive detection mode on a Waters Xevo G2-S Tof instrument equipped with an electron-spray ionization (ESI) source (Milford, MA).

### Synthesis

#### Preparation of 5-chloromethyl-8-quinolinol hydrochloride (2)

A mixture of 5.84 g (40.0 mmol) of 8-quinolinol, 50 ml of concentrated hydrochloric acid, and 6.4 ml of 37% formaldehyde was treated with 0.6 g of zinc chloride and stirred for 12 hours. The mixture was filtered, washed with copious acetone and dried to give compound **2** as a yellow solid (7.2 g, 78%). ^1^H NMR (400 MHz, deuterium oxide) δ 9.12 (dd, *J* = 8.7, 1.4 Hz, 1H), 8.88 (dd, *J* = 5.5, 1.4 Hz, 1H), 7.97 (dd, *J* = 8.7, 5.4 Hz, 1H), 7.59 (d, *J* = 8.0 Hz, 1H), 7.24 (d, *J* = 7.9 Hz, 1H), 4.93 (s, 2H).

#### General procedure for the synthesis of compounds (3a-c)

To a solution of substituted benzyl alcohol **3** (6 mmol) in anhydrous THF (30 mL) was added sodium hydride (60% dispersion in mineral oil, 0.72 g, 18 mmol) at 0°C. The suspension was stirred at 0°C for 30 minutes. Salt **2** (1.15 g, 5mmol) was added to the suspension. The mixture was stirred at r.t for 3 hours. Water was added to the suspension, and the mixture became homogeneous. The mixture was extracted by ethyl acetate and washed with brine, dried over anhydrous sodium sulfate and concentrated to get the crude. The crude compound was purified by flash chromatography (ethyl acetate: hexane 1:3)

#### 5-(((4-azidobenzyl)oxy)methyl)quinolin-8-ol (3a)

^1^HNMR (400 MHz, CDCl_3_) δ 8.78 (d, *J* = 2.8 Hz, 1H), 8.41 (d, *J* = 8.4 Hz, 1H), 7.45 (d, *J* = 8.0 Hz, 2H), 7.37 (d, *J* = 7.2 Hz, 1H), 7.24 (d, *J* = 8.0 Hz, 2H), 7.11 (d, *J* = 7.2 Hz, 1H), 4.84 (s, 2H), 4.52 (s, 2H).

#### 5-(((4-ethynylbenzyl)oxy)methyl)quinolin-8-ol (3b)

^1^HNMR (400 MHz, CDCl_3_) δ 8.77 (d, *J* = 2.8 Hz, 1H), 8.44 (d, *J* = 8.4 Hz, 1H), 7.45 (d, *J* = 8.0 Hz, 2H), 7.38 (d, *J* = 7.2 Hz, 1H), 7.25 (d, *J* = 8.0 Hz, 2H), 7.10 (d, *J* = 7.2 Hz, 1H), 4.84 (s, 2H), 4.50 (s, 2H), 3.07 (s, 3H).

#### 5-(((2-bromo-4-methylbenzyl)oxy)methyl)quinolin-8-ol (3c)

^1^H NMR (400 MHz, Chloroform-*d*) δ 8.85 (dd, *J* = 4.3, 1.6 Hz, 1H), 8.54 (dd, *J* = 8.5, 1.5 Hz, 1H), 7.60 – 7.51 (m, 2H), 7.46 (d, *J* = 7.8 Hz, 1H), 7.23 (d, *J* = 7.7 Hz, 1H), 7.17 (td, *J* = 5.0, 2.5 Hz, 2H), 4.89 (s, 2H), 4.50 (s, 2H), 2.41 (s, 3H).

#### General procedure for the synthesis of compounds (4a-c)

An equimolar mixture of the substrates **3**, paraformaldehyde, and the pyrrolidine in anhydrous ethanol (30 mL) was refluxed for 4 hours under argon. After cooling, the solvent was evaporated under reduced pressure. The crude compound was purified by flash chromatography (Dichloromethane: methanol 20: 1).

#### 5-(((4-azidobenzyl)oxy)methyl)-7- (pyrrolidin-1-ylmethyl)quinolin-8-ol (4a)

^1^HNMR (400 MHz, CDCl_3_) δ 8.89 (dd, *J* = 4, 1.6 Hz, 1H), 8.37 (dd, *J* = 8.4, 1.6 Hz, 1H), 7.41 (dd, *J* = 8.4, 4.0 Hz, 1H), 7.31 (d, *J* = 8.4 Hz, 2H), 7.21 (s, 1H), 7.00 (d, *J* = 8.4 Hz, 2H), 4.84 (s, 2H), 4.53 (s, 2H), 3.98 (S, 2H), 2.70 (m, 4H), 1.88 (m, 4H). HRMS (ESI): m/z calculated for C_22_H_23_N_5_O_2_ + H^+^ [M+ H]^+^: 390.1930; Found: 390.1942.

#### 5-(((4-ethynylbenzyl)oxy)methyl)-7- (pyrrolidin-1-ylmethyl)quinolin-8-ol (4b)

^1^HNMR (400 MHz, CDCl_3_) δ 8.88 (dd, *J* = 8.4, 1.6 Hz, 1H), 8.37 (dd, *J* = 8.4, 1.6 Hz, 1H), 7.46 (d, *J* = 8.4 Hz, 2H), 7.41 (dd, *J* = 8.4, 4.0 Hz, 1H), 7.28 (d, *J* = 8.4 Hz, 2H), 7.21 (s, 1H), 4.84 (s, 2H), 4.55 (s, 2H), 3.99 (s, 2H), 3.07 (s, 1H), 2.72-2.69 (m, 4H), 1.89-1.86 (m, 4H). HRMS (ESI): m/z calculated for C_24_H_24_N_2_O_2_ + H^+^ [M+ H]^+^: 373.1916; Found: 373.1924.

#### 5-(((2-bromo-4-methylbenzyl)oxy)methyl)-7- (pyrrolidin-1-ylmethyl)quinolin-8-ol (4c)

^1^H NMR (400 MHz, Chloroform-*d*) δ 8.79 (d, *J* = 4.1 Hz, 1H), 8.43 – 8.22 (m, 1H), 7.47 – 7.29 (m, 3H), 7.16 – 7.04 (m, 2H), 4.78 (s, 2H), 4.43 (s, 2H), 4.08 (s, 2H), 2.96 – 2.70 (m, 4H), 2.31 (s, 3H), 1.97 – 1.76 (m, 4H). Exact mass for C_23_H_25_BrN_2_O_2_: 442.1079; HRMS: [M+ H]^+^: 443.1170

#### Preparation of 5-((naphthalen-2-ylmethoxy)methyl)quinolin-8-ol (5)

To a solution of the alcohol in anhydrous DMF (10 mL) was added sodium hydride (60% dispersion in mineral oil, 0.72 g, 18 mmol) at 0°C. The suspension was stirred at 0°C for 30 minutes. Salt **2** (1.15 g, 5 mmol) was added to the suspension. The mixture was stirred at r.t for 3 hours. Water was added to the suspension, the mixture became homogeneous. The mixture was extracted by ethyl acetate and washed with brine, dried over anhydrous sodium sulfate and concentrated to get the crude. The crude compound was purified by flash chromatography (ethyl acetate: hexane 1:3) ^1^H NMR (400 MHz, Chloroform-*d*) δ 8.77 (dd, *J* = 4.2, 1.6 Hz, 1H), 8.38 (dd, *J* = 8.5, 1.6 Hz, 1H), 8.07 – 8.00 (m, 1H), 7.92 – 7.77 (m, 2H), 7.52 – 7.40 (m, 5H), 7.36 (dd, *J* = 8.5, 4.2 Hz, 1H), 7.11 (d, *J* = 7.7 Hz, 1H), 4.99 (s, 2H), 4.90 (s, 2H).

#### Preparation of 5-((naphthalen-2-ylmethoxy)methyl)-7- (pyrrolidin-1-ylmethyl)quinolin-8-ol (6)

An equimolar mixture of the substrates **5**, paraformaldehyde, and the pyrrolidine in anhydrous ethanol (30 mL) was refluxed for 4 hours under argon. After cooling, the solvent was evaporated under reduced pressure. The crude compound was purified by flash chromatography (Dichloromethane: methanol 20: 1). ^1^H NMR (400 MHz, Chloroform-*d*) δ 8.85 (dd, *J* = 4.2, 1.7 Hz, 1H), 8.30 (dd, *J* = 8.5, 1.6 Hz, 1H), 8.12 – 7.98 (m, 1H), 7.92 – 7.77 (m, 2H), 7.53 – 7.40 (m, 5H), 7.35 – 7.28 (m, 2H), 5.02 (s, 2H), 4.90 (s, 2H), 4.04 (s, 2H), 2.87 – 2.67 (m, 4H), 1.90 (p, *J* = 3.2 Hz, 4H). Exact mass for C_26_H_26_N_2_O_2_: 398.1994; HRMS: [M+ H]^+^: 399.2071

#### Preparation of 5- (morpholinomethyl)quinolin-8-ol (7)

To a solution of morpholine (6 mmol) in THF was added salt 2 (2 mmol). The mixture was refluxed for 3hr. The white precipitate was filtered, the filtrate was evaporated. The crude was purified by flash chromatography to produce **7**. ^1^H NMR (400 MHz, chloroform-*d*) δ 8.79 (dd, *J* = 4.2, 1.6 Hz, 1H), 8.67 (dd, *J* = 8.5, 1.6 Hz, 1H), 7.47 (dd, *J* = 8.5, 4.2 Hz, 1H), 7.33 (d, *J* = 7.7 Hz, 1H), 7.07 (d, *J* = 7.7 Hz, 1H), 3.79 (s, 2H), 3.70 – 3.61 (m, 4H), 2.45 (dd, *J* = 5.6, 3.7 Hz, 4H).

#### Preparation of 5- (morpholinomethyl)-7- (pyrrolidin-1-ylmethyl)quinolin-8-ol (8)

An equimolar mixture of the substrates **7**, paraformaldehyde, and the pyrrolidine in anhydrous ethanol (30 mL) was refluxed for 4 hours under argon. After cooling, the solvent was evaporated under reduced pressure. The crude compound was purified by flash chromatography (Dichloromethane: methanol 20: 1). ^1^H NMR (400 MHz, Chloroform-*d*) δ 8.86 (ddd, *J* = 5.9, 4.1, 1.6 Hz, 1H), 8.60 (td, *J* = 8.5, 1.7 Hz, 1H), 7.40 (dt, *J* = 8.4, 4.2 Hz, 1H), 7.19 (d, *J* = 29.0 Hz, 1H), 3.95 (d, *J* = 31.1 Hz, 2H), 3.86 (s, 1H), 3.81 – 3.74 (m, 3H), 3.66 (t, *J* = 4.7 Hz, 2H), 2.76 – 2.58 (m, 4H), 2.57 – 2.40 (m, 4H), 1.95 – 1.69 (m, 4H). Exact mass for C_19_H_25_N_3_O_2_: 327.1947; HRMS: [M+ H]^+^: 328.2026

#### Preparation of 2-chloroquinolin-8-ol (10)

Quinoline-2,8-diol (5.30 g, 32.89 mmol) was suspended in anhydrous DMF (30 mL). Thionyl chloride (9.45 mL, 131.50 mmol) was added dropwise *via* a syringe at 0°C under argon. The resulted solution was stirred and heated to 50°C for 4 hours. Then, the reaction was quenched by pouring the solution into ice water followed by extraction with ethyl acetate (3×50 mL), washed with brine (2×50 mL), dried over anhydrous MgSO_4_ and concentrated under reduced pressure and purified by silica gel column chromatography (eluting with CH_2_Cl_2_) to give a pale yellow solid product, 5.0 g, 84.7% yield.

#### Preparation of 2-chloro-5- (chloromethyl)quinolin-8-ol hydrochloride (11)

2-Chloroquinolin-8-ol (3.42 g, 19.04 mmol) was dissolved in 12N concentrated HCl (10 mL). 37% Formal aldehyde solution (8 mL) and ZnCl_2_ (1.10 g) were added. The reaction mixture was stirred at 40°C overnight. The precipitate was filtered and washed with copious acetone and dried under vacuum to give a yellow solid product, 3.25 g, 74.9% yield.

#### 2-chloro-5-(((4-isopropylbenzyl)oxy)methyl)quinolin-8-ol (12)

2-Chloro-5- (chloromethyl)quinolin-8-ol hydrochloride (0.55 g, 2.08 mmol) was suspended in 2 mL of 4-isopropylbenzyl alcohol. The reaction mixture was stirred and heated to 90°C under argon for 3 hours. The resulted solution was stirred in 10 mL of saturated NaHCO_3_ solution, extracted with ethyl acetate and washed with water. The extract was dried over anhydrous MgSO_4_ followed by filtration and concentration under reduced pressure. The residue was purified by silica gel column chromatography (eluting with CH_2_Cl_2_) to give a colorless oil product, 0.70 g, 98.5% yield. ^1^HNMR (400 MHz, CDCl_3_) δ 8.42 (d, *J* = 8.8 Hz, 1H), 7.77 (s, 1H), 7.41 (d, *J* = 8.8 Hz, 2H), 7.26 (s, 1H), 7.20 (d, *J* = 8.8 Hz, 2H), 7.13 (d, *J* = 8.0 Hz, 1H), 4.83 (s, 2H), 4.49 (s, 2H), 2.94-2.87 (m, 1H), 1.24 (d, *J* = 6.8 Hz, 6H).

#### Preparation of 2-chloro-5-(((4-isopropylbenzyl)oxy)methyl)-7- (pyrrolidin-1-ylmethyl)quinolin-8-ol (13)

2-chloro-5-(((4-isopropylbenzyl)oxy)methyl)quinolin-8-ol (0.50 g, 1.46 mmol), paraformaldehyde (44 mg, 1.46 mmol) and pyrrolidine (0.10 g, 1.46 mmol) were mixed together in 10 mL of ethanol. The reaction mixture was stirred and heated to reflux for 4 hours under argon. Then, the volatile was removed under reduced pressure. The residue was purified by silica gel column chromatography (eluting with CH_2_Cl_2_/MeOH= 9/1 v/v) to give a yellow solid product, 0.56 g, 90.3% yield. ^1^HNMR (400 MHz, CDCl_3_) δ 8.29 (d, *J* = 8.4 Hz, 1H), 7.35 (d, *J* = 8.4 Hz, 1H), 7.25 (d, *J* = 8.4 Hz, 2H), 7.21 (d, *J* = 8.4 Hz, 2H), 7.20 (s, 1H), 4.80 (s, 2H), 4.53 (s, 2H), 3.99 (s, 2H), 2.94-2.88 (m, 1H), 2.75-2.71 (m, 4H), 1.90-1.87 (m, 4H), 1.25 (d, *J* = 6.8 Hz, 6H). HRMS (ESI): m/z calculated for C_25_H_29_ClN_2_O_2_ + H^+^ [M+ H]^+^: 425.1996; Found: 425.1997.

#### Preparation of 5-(((4-Isopropylbenzyl)oxy)methyl)-2- (4-methylpiperazin-1-yl)quinolin-8-ol (14)

2-Chloro-5-(((4-isopropylbenzyl)oxy)methyl)quinolin-8-ol (0.20 g, 0.58 mmol) and (0.59 g, 5.85 mmol) were mixed together in 5 mL of pyridine. The reaction mixture was stirred and heated to reflux for 8 hours under argon. Then, the volatile was removed under reduced pressure. The residue was purified by silica gel column chromatography (eluting with CH_2_Cl_2_/MeOH= 9/1 v/v) to give a yellow solid product, 0.21 g, 87.5% yield. . ^1^HNMR (400 MHz, CDCl_3_) δ 8.21 (d, *J* = 9.2 Hz, 1H), 8.12 (s, 1H), 7.13 (d, *J* = 8.4 Hz, 2H), 7.08 (d, *J* = 8.4 Hz, 2H), 7.06 (s, 1H), 6.97 (d, *J* = 9.2 Hz, 1H), 4.69 (s, 2H), 4.36 (s, 2H), 4.74 (t, *J* = 4.8 Hz, 4H), 2.94-2.87 (m, 1H), 2.56 (t, *J* = 4.8 Hz, 4H), 2.37 (s, 3H), 2.07-2.05 (m, 4H), 1.20 (d, *J* = 6.8 Hz, 6H). HRMS (ESI): m/z calculated for C_25_H_31_N_3_O_2_ + H^+^ [M+ H]^+^: 406.2495; Found: 406.2485.

#### Preparation of 5-(((4-isopropylbenzyl)oxy)methyl)-2- (isopropylthio)-7- (pyrrolidin-1-ylmethyl)quinolin-8-ol (15)

Isopropyl thiol (0.21 g, 2.94 mmol) was dissolved in anhydrous THF (40 mL). NaH (60%wt in mineral oil, 0.14 g, 3.54 mmol) was added in portions at 0°C under argon. After stirred at room temperature for 2 hours, 2-Chloro-5-(((4-isopropylbenzyl)oxy)methyl)-7- (pyrrolidin-1-ylmethyl)quinolin-8-ol (**13**) (0.50 g, 1.18 mmol) was added. The resulted mixture was stirred at room temperature for 6 hours. Then, the reaction was quenched by addition of 50 mL of water. The mixture was extracted with CH_2_Cl_2_ (3×50 mL). The extracts was dried over anhydrous MgSO_4_, filtered and concentrated to dryness. The residue was purified by silica gel column chromatography (eluting with CH_2_Cl_2_/MeOH= 9/1 v/v) to give a yellow solid product, 0.46 mg, 83.9% yield. ^1^HNMR (400 MHz, CDCl_3_) δ 8.15 (d, *J* = 8.8 Hz, 1H), 7.29 (s, 1H), 7.23 (d, *J* = 8.8 Hz, 2H), 7.20 (d, *J* = 8.8 Hz, 2H), 7.19 (d, *J* = 8.0 Hz, 2H), 4.80 (s, 2H), 4.50 (s, 2H), 4.26-4.19 (m, 1H), 4.00 (s, 2H), 2.94-2.85 (m, 1H), 2.78-2.76 (m, 4H), 1.90-1.87 (m, 4H), 1.49 (d, *J* = 6.4 Hz, 6H), 1.24 (d, *J* = 6.8 Hz, 6H). HRMS (ESI): m/z calculated for C_28_H_36_N_2_O_2_S+ H^+^ [M+ H]^+^: 465.2576; Found: 465.2580.

#### Preparation of 5-(((4-isopropylbenzyl)oxy)methyl)-2- (4-methylpiperazin-1-yl)-7- (pyrrolidin-1-ylmethyl)quinolin-8-ol (16)

2-Chloro-5-(((4-isopropylbenzyl)oxy)methyl)-7- (pyridine-1-ylmethyl)quinolin-8-ol (**13**) (0.26 g, 0.76 mmol) and 4-methylpiperazine (0.76 g, 7.62 mmol) were mixed together in 5 mL of pyridine. The reaction mixture was stirred and heated to reflux for 2 hours under argon. Then, the volatile was removed under reduced pressure. The residue was purified by silica gel column chromatography (eluting with CH_2_Cl_2_/MeOH= 9/1 v/v) to give a yellow solid product, 0.17 g, 45.9% yield. ^1^HNMR (400 MHz, CDCl_3_) δ 8.15 (d, *J* = 9.2 Hz, 1H), 7.25 (d, *J* = 8.0 Hz, 2H), 7.20 (d, *J* = 8.0 Hz, 2H), 7.03 (s, 1H), 6.74 (d, *J* = 9.2 Hz, 1H), 4.77 (s, 2H), 4.47 (s, 2H), 3.83 (s, 2H), 3.60 (t, *J* = 6.4 Hz, 4H), 2.94-2.87 (m, 1H), 2.75-2.72 (m, 4H), 2.67-2.64 (m, 4H), 2.38 (s, 3H), 2.07-2.05 (m, 4H), 1.24 (d, *J* = 6.8 Hz, 6H). HRMS (ESI): m/z calculated for C_30_H_40_N_4_O_2_ + H^+^ [M+ H]^+^: 489.3230; Found: 489.3236.

#### Preparation of 7((1H-imidazol-1-yl)methyl-2-chloro-5-(((4-isopropylbenzyl)oxy)methyl)quinolin-8-ol (17)

2-Chloro-5-(((4-isopropylbenzyl)oxy)methyl)-7- (pyrrolidin-1-ylmethyl)quinolin-8-ol (**13**) (0.12 g, 0.28 mmol) and imidazole (0.19 g, 2.82 mmol) were mixed together in anhydrous pyridine (5 mL) under argon. The reaction mixture was stirred and heated to reflux for 6 hours under argon. Then, the volatile was removed under reduced pressure. The residue was purified by silica gel column chromatography (eluting with CH_2_Cl_2_/MeOH= 19/1 v/v) to give a yellow solid product, 65 mg, 54.6% yield. ^1^HNMR (400 MHz, CDCl_3_) δ 8.56 (s, H), 8.41 (d, *J* = 9.2 Hz, 1H), 7.48 (d, *J* = 8.8 Hz, 1H), 7.25-7.23 (m, 6H), 5.51 (s, 2H), 4.82 (s, 2H), 2.96-2.89 (m, 1H), 2.16 (s, 2H), 1.26 (d, *J* = 7.2 Hz, 6H). HRMS (ESI): m/z calculated for C_24_H_24_ClN_3_O_2_ + H^+^ [M+ H]^+^: 422.1635; Found: 422.1648.

### Procedure for the preparation of compound 22

#### 2-Hydroxyquinolin-8-yl acetate (18)

Quinoline-2,8-diol (5.00 g, 31.00 mmol) was dissolved in anhydrous THF (30 mL) at room temperature under argon. Acetic anhydride (10 mL) was added *via* a syringe. The reaction solution was stirred and heated to reflux overnight. Then, the volatile was removed under reduced pressure. The residue was purified by silica gel column chromatography (eluting with CH_2_Cl_2_/MeOH= 19/1 v/v) to give a white solid product, 5.80 g, 92.1% yield.

#### 2-Bromoquinolin-8-yl acetate (19)

2-Hydroxyquinolin-8-yl acetate (2.50 g, 12.30 mmol) was dissolved in chloroform (10 mL). POBr_3_ (8.82 g, 30.75 mmol) was added at room temperature. The reaction mixture was stirred and heated to reflux for 6 hours under argon. The reaction solution was cooled to room temperature and poured into ice water followed by extraction with CH_2_Cl_2_, dried over anhydrous MgSO_4_/K_2_CO_3_, filtration and concentrated under reduced pressure. The residue was purified by silica gel column chromatography (eluting with CH_2_Cl_2_) to give a white solid product, 2.80 g, 86.0% yield. ^1^HNMR (400 MHz, CDCl_3_) δ 8.97 (d, *J* = 8.4 Hz, 1H), 7.69 (dd, *J* = 8.4, 1.2 Hz, 1H), 7.57-7.52 (m, 2H), 7.46 (dd, *J* = 8.4, 1.2 Hz, 1H), 2.50 (s, 3H).

#### 2-Bromo-5- (chloromethyl)quinolin-8-ol hydrochloride (20)

2-Bromoquinolin-8-yl acetate (1.20 g, 4.51 mmol) was suspended in concentrated HCl (10 mL) at room temperature. ZnCl_2_ (0.40 g) was added. The reaction mixture was stirred and heated to 40°C for 8 hours. The yellow precipitate was separated, washed with acetone and dried under vacuum to give a yellow solid product, 0.92 g, 66.0% yield. ^1^HNMR (400 MHz, DMSO-*d*^6^) δ 8.46 (d, *J* = 7.2 Hz, 1H), 7.79 (d, *J* = 8.8 Hz, 1H), 7.61 (d, *J* = 8.0 Hz, 1H), 7.11 (d, *J* = 8.0 Hz, 1H), 5.19 (s, 2H).

#### 2-Bromo-5-(((4-isopropylbenzyl)oxy)methyl)quinolin-8-ol (21)

2-Bromo-5- (chloromethyl)quinolin-8-ol hydrochloride (0.50 g, 1.62 mmol) was suspended in 5 mL of 4-isopropylbenzyl alcohol. The reaction mixture was stirred and heated to 90°C under argon for 4 hours. The resulted solution was stirred in 10 mL of saturated NaHCO_3_ solution, extracted with ethyl acetate and washed with water. The extract was dried over anhydrous MgSO_4_ followed by filtration and concentration under reduced pressure. The residue was purified by silica gel column chromatography (eluting with CH_2_Cl_2_) to give a colorless oil product, 0.58 g, 92.7% yield. ^1^HNMR (400 MHz, CDCl_3_) δ 8.41 (d, *J* = 8.8 Hz, 1H), 7.77 (s, 1H), 7.41 (d, *J* = 8.8 Hz, 2H), 7.24 (d, *J* = 8.8 Hz, 1H), 7.20 (d, *J* = 8.8 Hz, 2H), 4.84 (s, 2H), 4.40 (s, 2H), 2.94-2.87 (m, 1H), 1.24 (d, *J* = 7.2 Hz, 6H).

#### 2-Bromo-5-(((4-isopropylbenzyl)oxy)methyl)-7- (pyrrolidin-1-ylmethyl)quinolin-8-ol (22)

2-Bromo-5-(((4-isopropylbenzyl)oxy)methyl)quinolin-8-ol (0.84 g, 2.17 mmol), paraformaldehyde (65 mg, 2.17 mmol) and pyrrolidine (0.16 g, 2.17 mmol) were mixed together in 10 mL of ethanol. The reaction mixture was stirred and heated to reflux for 4 hours under argon. Then, the volatile was removed under reduced pressure. The residue was purified by silica gel column chromatography (eluting with CH_2_Cl_2_/MeOH= 9/1 v/v) to give a yellow solid product, 0.74 g, 72.6% yield. ^1^HNMR (400 MHz, CDCl_3_) δ 8.30 (d, *J* = 8.8 Hz, 1H), 7.36 (d, *J* = 8.8 Hz, 1H), 7.25 (d, *J* = 8.4 Hz, 2H), 7.21 (d, *J* = 8.4 Hz, 2H), 7.23 (s, 1H), 4.80 (s, 2H), 4.53 (s, 2H), 4.01 (s, 2H), 2.94-2.87 (m, 1H), 2.74-2.72 (m, 4H), 1.91-1.88 (m, 4H), 1.24 (d, *J* = 6.8 Hz, 6H). HRMS (ESI): m/z calculated for C_25_H_29_BrN_2_O_2_ + H^+^ [M+ H]^+^: 469.1491; Found: 469.1490.

### Synthesis of 4-((tert-butyldimethylsilyl)oxy)-1-naphthaldehyde (24)

To a solution of aldehyde 15 (2 mmol) in DCM was added TBDMSCl (3 mmol) and imidazole (4 mmol). The mixture was stirred overnight. Water was added and the mixture was extracted with DCM. The organic layer was dried over anhydrous sodium sulfate. The crude was purified by flash chromatography to generate **24**. ^1^H NMR (400 MHz, Chloroform-*d*) δ 10.12 (s, 1H), 9.21 (dt, *J* = 8.7, 1.0 Hz, 1H), 8.18 (ddd, *J* = 8.5, 1.5, 0.7 Hz, 1H), 7.77 (d, *J* = 7.9 Hz, 1H), 7.59 (ddd, *J* = 8.5, 6.9, 1.5 Hz, 1H), 7.48 (ddd, *J* = 8.3, 6.9, 1.2 Hz, 1H), 6.85 (d, *J* = 7.9 Hz, 1H), 1.01 (s, 9H), 0.27 (s, 6H).

### Synthesis of (4-((tert-butyldimethylsilyl)oxy)naphthalen-1-yl)methanol (25)

To the protected aldehyde 16 (1 mmol) in methanol was added NaBH4 (3 mmol). The mixture was stirred at room temperature for 2 hours. The solvent was evaporated and extracted with ethyl acetate. The crude was subject to flash chromatography to produce alcohol 25. ^1^H NMR (400 MHz, Chloroform-*d*) δ 8.24 (ddd, *J* = 8.1, 1.5, 0.7 Hz, 1H), 8.16 – 8.03 (m, 1H), 7.52 (dddd, *J* = 22.0, 8.1, 6.8, 1.4 Hz, 2H), 7.34 (d, *J* = 7.6 Hz, 1H), 6.81 (d, *J* = 7.7 Hz, 1H), 5.06 (s, 2H), 1.10 (s, 9H), 0.29 (s, 6H).

### Synthesis of((4-((benzyloxy)methyl)naphthalen-1-yl)oxy) (tert-butyl)dimethylsilane (26)

To a solution of alcohol **25** in anhydrous THF (10ml) was added sodium hydride (60% dispersion in mineral oil) at 0°C. The suspension was stirred at 0°C for 30 minutes. Benzyl bromide was added to the suspension. The mixture was stirred at r.t for 3 hours. Water was added to the suspension, the mixture became homogeneous. The mixture was extracted by ethyl acetate and washed with brine, dried over anhydrous sodium sulfate and concentrated to get the crude. The crude compound was purified by flash chromatography (ethyl acetate: hexane 1:3). ^1^H NMR (400 MHz, Chloroform-*d*) δ 8.31 – 8.24 (m, 1H), 7.92 – 7.82 (m, 1H), 7.45 – 7.34 (m, 3H), 7.34 – 7.27 (m, 3H), 7.26 – 7.19 (m, 2H), 6.73 (d, *J* = 7.9 Hz, 1H), 5.13 (s, 2H), 5.00 (s, 2H), 0.83 (s, 9H), 0.00 (s, 6H).

### Synthesis of 4-((benzyloxy)methyl)naphthalen-1-ol (27)

To a solution of alcohol 18 in THF was added TBAF (1.5 eq) and stirred for 1 hour. The mixture was extracted by ethyl acetate. The crude was subject to flash chromatography to produce phenol **27**. ^1^H NMR (400 MHz, DMSO-*d*_6_) δ 10.21 (s, 1H), 8.18 (ddd, *J* = 8.2, 1.6, 0.7 Hz, 1H), 8.02 (ddd, *J* = 8.4, 1.3, 0.7 Hz, 1H), 7.58 – 7.38 (m, 3H), 7.36 – 7.25 (m, 5H), 6.82 (d, *J* = 7.6 Hz, 1H), 4.84 (s, 2H), 4.54 (s, 2H).

### Synthesis of 4-((benzyloxy)methyl)-2- (pyrrolidin-1-ylmethyl)naphthalen-1-ol (28)

An equimolar mixture of the substrates **27**, paraformaldehyde, and the pyrrolidine in anhydrous ethanol (30 mL) was refluxed for 4 hours under argon. After cooling, the solvent was evaporated under reduced pressure. The crude compound was purified by flash chromatography (Dichloromethane: methanol 20: 1). ^1^H NMR (400 MHz, Chloroform-*d*) δ 8.31 – 8.23 (m, 1H), 8.05 – 7.94 (m, 1H), 7.57 – 7.44 (m, 2H), 7.42 – 7.26 (m, 5H), 7.10 (s, 1H), 4.88 (s, 2H), 4.61 (s, 2H), 3.99 (s, 2H), 2.89 – 2.52 (m, 4H), 2.00 – 1.77 (m, 4H). Exact mass for C_23_H_25_NO_2_: 347.1885; HRMS: [M+ H]^+^: 348.1959

### Synthesis of 1- (benzyloxy)-4-((benzyloxy)methyl)benzene (30)

To a solution of alcohol **29** (3 mmol) in anhydrous THF (30ml) was added sodium hydride (60% dispersion in mineral oil, 6 mmol) at 0°C. The suspension was stirred at 0°C for 30 minutes. Benzyl bromide (4 mmol) was added to the suspension. The mixture was stirred at r.t for 3 hours. Water was added to the suspension, the mixture became homogeneous. The mixture was extracted by ethyl acetate and washed with brine, dried over anhydrous sodium sulfate and concentrated to get the crude. The crude compound was purified by flash chromatography (ethyl acetate: hexane 1:3). ^1^H NMR (400 MHz, Chloroform-*d*) δ 7.52 – 7.18 (m, 12H), 7.03 – 6.83 (m, 2H), 5.07 (s, 2H), 4.53 (s, 2H), 4.49 (s, 2H).

### Synthesis of 4-((benzyloxy)methyl)phenol (31)

To a solution of intermediate 12 (1.0 equiv.) and NiCl_2_.6H_2_O (1.5 equiv.) in methanol (5 mL) at 0°C, NaBH_4_ (3.0 equiv.) was added and stirred until complete consumption of the starting material. The progress of the reaction was monitored by TLC. After completion, the reaction mixture was quenched with methanol and stirred for another 20 minutes. It was then filtered through celite pad and the filtrate was concentrated under reduced pressure. The crude product, thus obtained, was purified by column chromatography on activated silica gel using ethyl acetate-hexane mixture as eluent to afford the pure phenol **13**. ^1^H NMR (400 MHz, Chloroform-*d*) δ 7.39 – 7.33 (m, 4H), 7.32 – 7.27 (m, 1H), 7.26 – 7.21 (m, 2H), 6.82 – 6.75 (m, 2H), 4.54 (s, 2H), 4.47 (s, 2H).

### Synthesis for 4-((benzyloxy)methyl)-2- (pyrrolidin-1-ylmethyl)phenol (32)

An equimolar mixture of the substrates **13**, paraformaldehyde, and the pyrrolidine in anhydrous ethanol (30 mL) was refluxed for 4 hours under argon. After cooling, the solvent was evaporated under reduced pressure. The crude compound was purified by flash chromatography (Dichloromethane: methanol 20: 1). ^1^H NMR (400 MHz, Chloroform-*d*) δ 7.39 – 7.33 (m, 4H), 7.31 – 7.27 (m, 1H), 7.13 (dd, *J* = 8.2, 2.2 Hz, 1H), 6.99 (d, *J* = 2.2 Hz, 1H), 6.79 (d, *J* = 8.2 Hz, 1H), 4.54 (s, 2H), 4.43 (s, 2H), 3.82 (s, 2H), 2.71 – 2.53 (m, 4H), 1.91 – 1.80 (m, 4H). Exact mass for C_19_H_23_NO_2_: 297.1729; HRMS: [M+ H]^+^: 298.1812

### Synthesis of 5-(((4-isopropylbenzyl)oxy)methyl)-7- (pyrrolidin-1-ylmethyl)quinolin-8-yl trifluoromethanesulfonate (33)

To a solution of MX106 in anhydrous THF was added sodium hydride at 0°C. The mixture was added at 0°C for 30 minutes. N-Phenyl-bis (trifluoromethanesulfonimide) was added. The mixture was refluxed for 4hr. The mixture was extracted by ethyl acetate. The crude was subject to flash chromatography to generate **33**. ^1^H NMR (400 MHz, Chloroform-*d*) δ 9.00 (dd, *J* = 4.2, 1.6 Hz, 1H), 8.47 (dd, *J* = 8.6, 1.6 Hz, 1H), 7.84 (s, 1H), 7.51 (dd, *J* = 8.5, 4.2 Hz, 1H), 7.29 (d, *J* = 8.2 Hz, 2H), 7.24 (d, *J* = 8.2 Hz, 2H), 4.95 (s, 2H), 4.59 (s, 2H), 3.95 (s, 2H), 2.93 (p, *J* = 6.9 Hz, 1H), 2.74 – 2.44 (m, 4H), 1.96 – 1.71 (m, 4H), 1.26 (d, *J* = 6.9 Hz, 6H). Exact mass for C_26_H_29_F_3_N_2_O_4_S: 522.1800; HRMS: [M+ H]^+^: 523.1884

### Synthesis of 5-(((4-isopropylbenzyl)oxy)methyl)-7- (pyrrolidin-1-ylmethyl)quinoline (34)

To a solution of compound **33** in methanol was added Pd/c (10%, 0.05eq), Mg (0.1 eq) and ammonium acetate (2 eq). The mixture was stirred at room temperate for 5 hours. The mixture was filtered by celite. The crude was purified to generate compound **25**. ^1^H NMR (400 MHz, Chloroform-*d*) δ 8.93 – 8.77 (m, 1H), 8.59 – 8.45 (m, 1H), 8.07 (s, 1H), 7.82 (d, *J* = 1.7 Hz, 1H), 7.46 (dd, *J* = 8.6, 4.3 Hz, 1H), 7.20 (d, *J* = 7.5 Hz, 3H), 7.13 (d, *J* = 8.2 Hz, 2H), 4.91 (s, 2H), 4.52 (s, 2H), 4.41 (s, 2H), 3.57 (s, 2H), 3.06 (s, 2H), 2.82 (p, *J* = 6.9 Hz, 1H), 2.08 (s, 5H), 1.16 (d, *J* = 6.9 Hz, 6H). Exact mass for C_25_H_30_N_2_O: 374.2358; HRMS: [M+ H]^+^: 375.2439

### Synthesis for 5-(((4-isopropylbenzyl)oxy)methyl)-7- (pyrrolidin-1-ylmethyl)-1,2,3,4-tetrahydroquinoline (35)

To a solution of compound **33** in methanol was added Pd/C (10%, 0.1eq), Mg (0.2 eq) and ammonium acetate (2 eq). The mixture was stirred at room temperate overnight. The mixture was filtered by celite. The crude was purified to generate compound 35. ^1^H NMR (400 MHz, Chloroform-*d*) δ 9.95 (s, 1H), 7.21 (d, *J* = 8.2 Hz, 2H), 7.17 – 7.13 (m, 2H), 6.67 (d, *J* = 1.9 Hz, 1H), 6.52 (d, *J* = 1.9 Hz, 1H), 4.46 (s, 2H), 4.37 (s, 2H), 3.96 (s, 2H), 3.56 (s, 3H), 3.24 – 3.12 (m, 2H), 2.94 – 2.74 (m, 2H), 2.60 (t, *J* = 6.5 Hz, 2H), 2.11 (d, *J* = 8.5 Hz, 2H), 1.96 (d, *J* = 7.9 Hz, 2H), 1.84 (td, *J* = 6.4, 4.6 Hz, 2H), 1.18 (d, *J* = 6.9 Hz, 6H). Exact mass for C_25_H_34_N_2_O: 378.2671; HRMS: [M+ H]^+^: 379.2750

### General procedure for the synthesis of compounds (36a-b)

To a solution of alcohol (6 mmol) in anhydrous THF (30ml) was added sodium hydride (60% dispersion in mineral oil, 0.72 g, 18 mmol) at 0°C. The suspension was stirred at 0°C for 30 minutes. Salt **2** (1.15 g, 5mmol) was added to the suspension. The mixture was stirred at r.t for 3 hours. Water was added to the suspension, the mixture became homogeneous. The mixture was extracted by ethyl acetate and washed with brine, dried over anhydrous sodium sulfate and concentrated to get the crude. The crude compound was purified by flash chromatography (ethyl acetate: hexane 1:3)

####  (R)-5-((1- (2,6-dichloro-3-fluorophenyl)ethoxy)methyl)quinolin-8-ol (36a)

^1^H NMR (400 MHz, Chloroform-*d*) δ 8.83 (dd, *J* = 4.3, 1.6 Hz, 1H), 8.59 (dd, *J* = 8.6, 1.6 Hz, 1H), 7.54 (dd, *J* = 8.5, 4.3 Hz, 1H), 7.35 (d, *J* = 7.7 Hz, 1H), 7.28 – 7.25 (m, 1H), 7.12 (d, *J* = 7.7 Hz, 1H), 7.05 (ddd, *J* = 8.9, 8.0, 4.6 Hz, 1H), 5.34 (q, *J* = 6.8 Hz, 1H), 4.85 (d, *J* = 11.8 Hz, 1H), 4.66 (d, *J* = 11.8 Hz, 1H), 1.60 (d, *J* = 6.8 Hz, 3H).

#### (S)-5-((1- (2,6-dichloro-3-fluorophenyl)ethoxy)methyl)quinolin-8-ol (36b)

^1^H NMR (400 MHz, Chloroform-*d*) δ 8.78 (dd, *J* = 4.2, 1.6 Hz, 1H), 8.52 (dd, *J* = 8.5, 1.5 Hz, 1H), 7.49 (dd, *J* = 8.5, 4.2 Hz, 1H), 7.33 – 7.27 (m, 1H), 7.25 – 7.22 (m, 1H), 7.07 – 7.04 (m, 1H), 7.03 – 6.99 (m, 1H), 5.58 (s, 1H), 5.31 (q, *J* = 6.8 Hz, 1H), 4.82 (dd, *J* = 11.7, 0.6 Hz, 1H), 4.62 (d, *J* = 11.8 Hz, 1H), 1.65 (d, *J* = 6.9 Hz, 3H).

### General procedure for the synthesis of compounds (37a-b)

An equimolar mixture of the substrates **36**, paraformaldehyde, and the pyrrolidine in anhydrous ethanol (30 mL) was refluxed for 4 hours under argon. After cooling, the solvent was evaporated under reduced pressure. The crude compound was purified by flash chromatography (Dichloromethane: methanol 20: 1).

####  (R)-5-((1- (2,6-dichloro-3-fluorophenyl)ethoxy)methyl)-7- (pyrrolidin-1-ylmethyl)quinolin-8-ol (37a)

^1^H NMR (400 MHz, Chloroform-*d*) δ 8.79 (dd, *J* = 4.2, 1.7 Hz, 1H), 8.38 (dd, *J* = 8.5, 1.7 Hz, 1H), 7.35 (dd, *J* = 8.5, 4.2 Hz, 1H), 7.13 (dd, *J* = 8.9, 4.9 Hz, 1H), 7.05 (s, 1H), 6.92 (dd, *J* = 8.9, 8.0 Hz, 1H), 5.24 – 5.16 (m, 2H), 4.73 (d, *J* = 11.8 Hz, 1H), 4.56 (d, *J* = 11.8 Hz, 1H), 3.92 (d, *J* = 1.6 Hz, 2H), 2.76 – 2.55 (m, 4H), 1.88 – 1.73 (m, 4H), 1.50 (d, *J* = 6.8 Hz, 3H). Exact mass for C_23_H_23_Cl_2_FN_2_O_2_: 448.1121; HRMS: [M+ H]^+^: 449.1191

####  (S)-5-((1- (2,6-dichloro-3-fluorophenyl)ethoxy)methyl)-7- (pyrrolidin-1-ylmethyl)quinolin-8-ol (37b)

^1^H NMR (400 MHz, Chloroform-*d*) δ 8.87 (dd, *J* = 4.1, 1.6 Hz, 1H), 8.45 (dd, *J* = 8.5, 1.7 Hz, 1H), 7.41 (dd, *J* = 8.5, 4.2 Hz, 1H), 7.20 (dd, *J* = 8.8, 4.9 Hz, 1H), 7.09 (s, 1H), 6.99 (dd, *J* = 8.9, 8.0 Hz, 1H), 5.35 – 5.22 (m, 2H), 4.80 (d, *J* = 11.8 Hz, 1H), 4.62 (d, *J* = 11.8 Hz, 1H), 3.97 (s, 2H), 2.81 – 2.59 (m, 4H), 1.95 – 1.77 (m, 4H), 1.57 (d, *J* = 6.8 Hz, 3H). Exact mass for C_23_H_23_Cl_2_FN_2_O_2_: 448.1121; HRMS: [M+ H]^+^: 449.1205

### Synthesis of 5-(((4-trifluoromethylbenzyl)amino)methyl)quinolin-8-ol (38)

5- (Chloromethyl)quinolin-8-ol hydrochloride (0.50 g, 2.17 mmol) and 4-trifluorobenzylamine (1.14 g, 6.52 mmol) were mixed together in 40 mL of ethyl acetate/DMF (1/3). The reaction mixture was stirred and heated to 60°C for 24 hours. The reaction was quenched by addition of 50 mL of saturated sodium bicarbonate solution and extracted with CH_2_Cl_2_ (3×50 mL). The extracts was dried over anhydrous MgSO_4_ followed by filtration and concentration under reduced pressure. The residue was purified by silica gel column chromatography (eluting with CH_2_Cl_2_/MeOH 19/1 v/v) to give a gray solid, 0.26 g, 36.1% yield. ^1^HNMR (400 MHz, CDCl_3_) δ 8.81 (d, *J* = 3.2 Hz, 1H), 8.52 (d, *J* = 8.4 Hz, 1H), 7.61 (d, *J* = 8.0 Hz, 2H), 7.49 (d, *J* = 8.0 Hz, 2H), 7.41 (d, *J* = 8.0 Hz, 1H), 7.11 (d, *J* = 8.0 Hz, 1H),4.16 (s, 2H), 3.95 (s, 2H).

### Synthesis of 2-bromo-5-(((4-isopropylbenzyl)oxy)methyl)-7- (pyrrolidin-1-ylmethyl)quinolin-8-ol. (39)

5-(((4-trifluoromethylbenzyl)amino)methyl)quinolin-8-ol (0.16 g, 0.48 mmol), para-formaldehyde (14 mg, 0.48 mmol) and pyrrolidine (34 mg, 0.48 mmol) were mixed together in 10 mL of ethanol. The reaction mixture was stirred and heated to reflux for 4 hours under argon. Then, the volatile was removed under reduced pressure. The residue was purified by silica gel column chromatography (eluting with CH_2_Cl_2_/MeOH= 9/1 v/v) to give a yellow solid product, 0.14 g, 70.4% yield. ^1^HNMR (400 MHz, CDCl_3_) δ 8.79 (d, *J* = 3.6 Hz, 1H), 8.01 (dd, *J* = 8.4, 1.6 Hz, 1H), 7.50 (d, *J* = 7.2 Hz, 1H), 7.31 (d, *J* = 8.4 Hz, 2H), 7.27 (d, *J* = 8.4 Hz, 2H), 7.15 (s, 1H), 3.96 (s, 2H), 3.57 (s, 2H), 3.28 (s, 2H), 2.68-2.65 (m, 4H), 1.82-1.80 (m, 4H). HRMS (ESI): m/z calculated for C_23_H_24_F_3_N_3_O+ H^+^ [M+ H]^+^: 416.1950; Found: 416.1965.

### Cell culture and ***in vitro*** antiproliferative assays in human melanoma cells

Human melanoma A375 and M14 cell lines were purchased from ATCC (American Type Culture Collection, Manassas, VA, USA), and cultured in DMEM media (Mediatech, Inc., Manassas, VA) at 37°C in a humidified atmosphere containing 5% CO_2_. The culture media were supplemented with 10% fetal bovine serum (Atlanta Biologicals, Lawrenceville, GA) and 1% antibiotic-antimycotic mixture (Sigma-Aldrich, St. Louis, MO). Compounds were dissolved in dimethylsulfoxide (DMSO; Sigma-Aldrich) to make a stock solution of 10 mmol/L. Compound solutions were freshly prepared by diluting stocks with cell culture medium before use (final solution contained less than 0.5% DMSO). Five thousand cells in logarithm growing phase were seeded overnight into each well of a 96-well plate. Then the cells were continuously incubated for 48 hours with sequential diluted compound solution (3 nmol/L to 100 μmol/L, 100 μL per well) in cell culture medium. The cell viability was determined in MTS assay and IC_50_ was calculated (*n* = 4), following similar procedures as described previously^[25^–^28]^.

### Cell culture and cytotoxicity assays in human epidermoid carcinoma and colorectal cancer cells

Dulbecco’s modified Eagle's Medium (DMEM), fetal bovine serum (FBS), penicillin/streptomycin and trypsin 0.25% were purchased from Hyclone (GE Healthcare Life Science, Pittsburgh, PA). Phosphate buffered saline (PBS) was purchased from Invitrogen GIBCO (Grand Island, NY). Dimethyl sulfoxide (DMSO) and 3- (4,5-dimethylthiazole-2-yl)-2,5-biphenyl tetrazolium bromide (MTT) were purchased from Sigma Chemical Co (St. Louis, MO).

The P-glycoprotein (P-gp) overexpressing KB-C2 cell line was established from a parental human epidermoid carcinoma cell line KB-3-1, by a step-wise selection of KB-3-1 in increasing concentrations of colchicine up to 2 μg/mL^[29]^. SW620/Ad300, which is also a P-gp overexpressing drug resistant cell line, was established by stepwise exposure of the parental human colon cancer cell line SW620 to increasing concentrations of doxorubicin up to 300 ng/mL^[30]^. The SW620 and SW620/Ad300 cell lines were kindly provided by Dr. Susan E. Bates and Dr. Robert W. Robey (NCI, NIH, Bethesda, MD, USA). All the cell lines were grown in DMEM supplemented with 10% FBS and 100 unit/mL penicillin/streptomycin in a humidified incubator containing 5% CO_2_ at 37°C.

The MTT colorimetric assay was used to measure the sensitivity of the cells against the synthesized compounds. The assay detects the formazan product formed from the reduction of MTT in active cells thus assesses the cell viability^[31]^. Cells were seeded in 96-well plates at 5,000 cells/well (KB-3-1 or KB-C2 cells) or at 7000 cells/well (SW620 or SW620/Ad300 cells) in 180 μL completed medium and cultured overnight. Then various concentrations of the compounds (20 μL) were added to the designated wells. After 72 hours continuous drug incubation, 20 μL of MTT reagent (4 mg/mL) was added to each well and the plates were incubated at 37°C for 4 hours. Subsequently, the medium was removed and 100 µL of DMSO were added to dissolve the formazan crystals in each well. The absorbance was determined at 570 nm by the accuSkan^TM^ GO UV/Vis Microplate Spectrophotometer (Fisher Sci., Fair Lawn, NJ). The IC_50_ values of each compound on each cell line were calculated from the survival curves to represent the cytotoxicity of the compounds. The fold of drug resistance was calculated by dividing the IC_50_ of the P-gp overexpressing cells by that of the parental cells. Two known P-gp substrates, YM155 and paclitaxel, were used as positive controls for P-gp overexpressing cell lines. On the other hand, cisplatin, which is not a substrate of P-gp, was used as negative control drug.

### Western blotting

To determine the change of protein levels of survivin and closely related IAPs, lysates of A375 or M14 melanoma cells treated by the compound solution for 24 hours were used for Western blotting analysis. Primary rabbit antibodies against IAP proteins including survivin (#2808), XIAP (#2045), cIAP1 (#7065), Livin (#5471), Cleaved PARP (#9185) and the loading control protein GAPDH (HRP Conjugate) (#3683) were purchased from Cell Signaling Technology, Inc. (Danvers, MA) and used according to manufacture instructions as reported previously. Protein lane intensities were quantified using ImageJ software (US National Institutes of Health, Bethesda, MD) and band intensities were normalized with vehicle control and GAPDH loading control.

### Statistical analysis

All experiments were repeated at least three times and the differences between the mean values of two groups were determined by using two-tailed Student's *t*-test in cell cytotoxicity assay. The difference between the survival curves of two groups were determined by two-way ANOVA. Results were considered statistically significant when *P*<0.05.

## Results

### Chemistry

**Fig.2:**
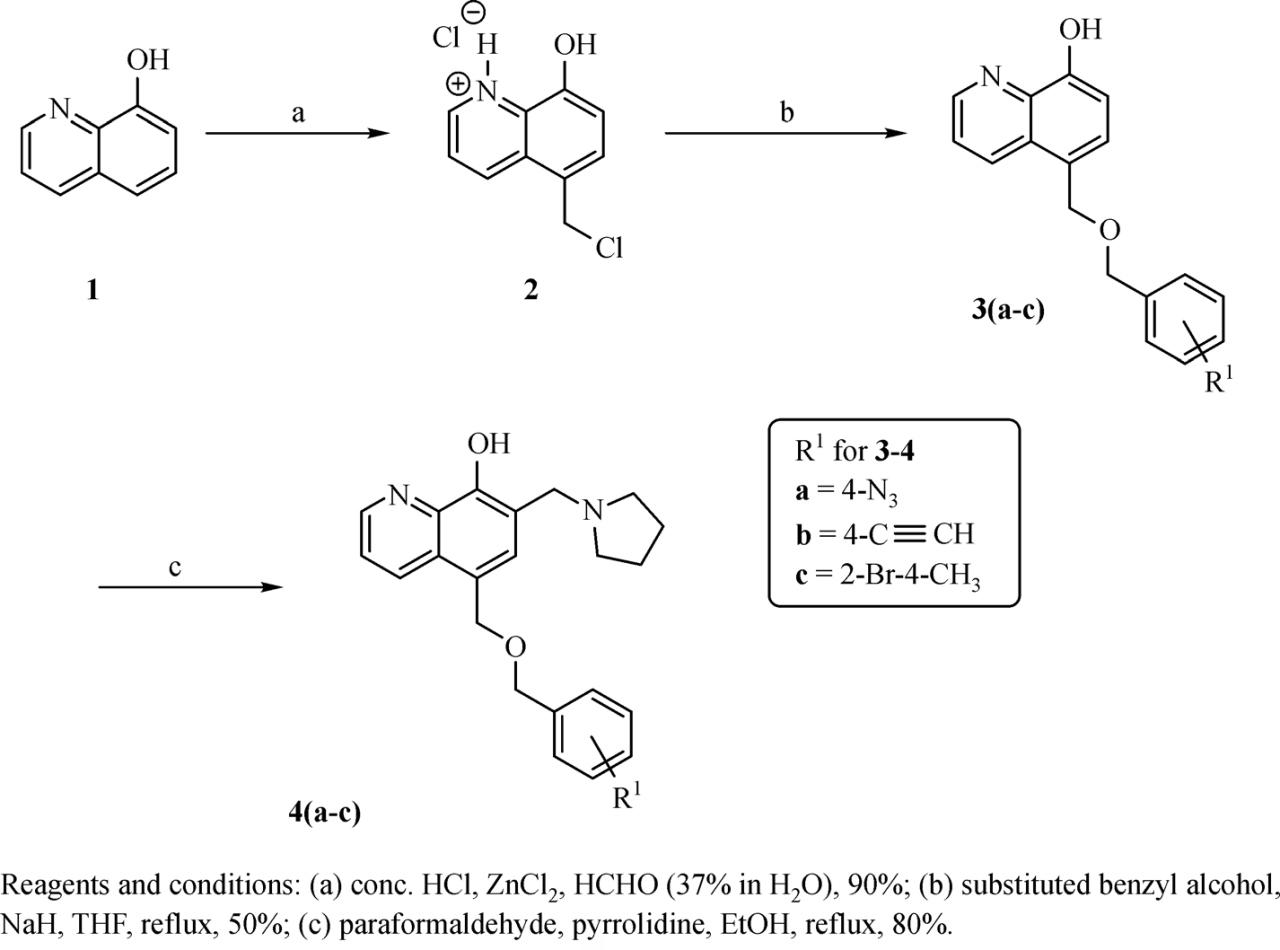
Synthesis of Compounds **4a-4c**

**Fig.3:**
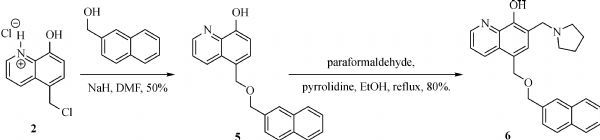
Synthesis of Compound **6**

**Fig.4:**
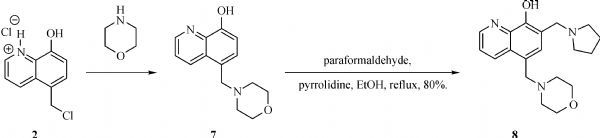
Synthesis of Compound **8**

**Fig.5:**
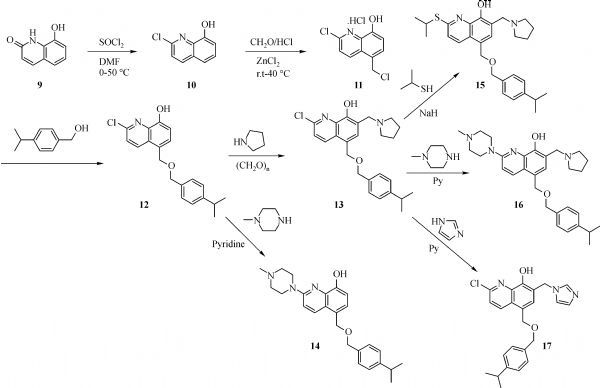
Synthesis of Compounds **13-17**

**Fig.6:**
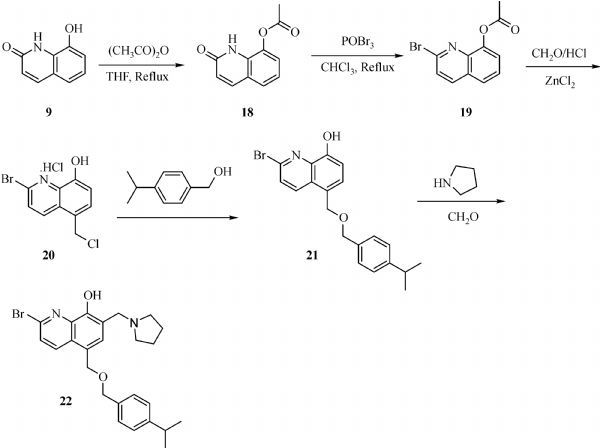
Synthesis of Compound **22**

**Fig.7:**
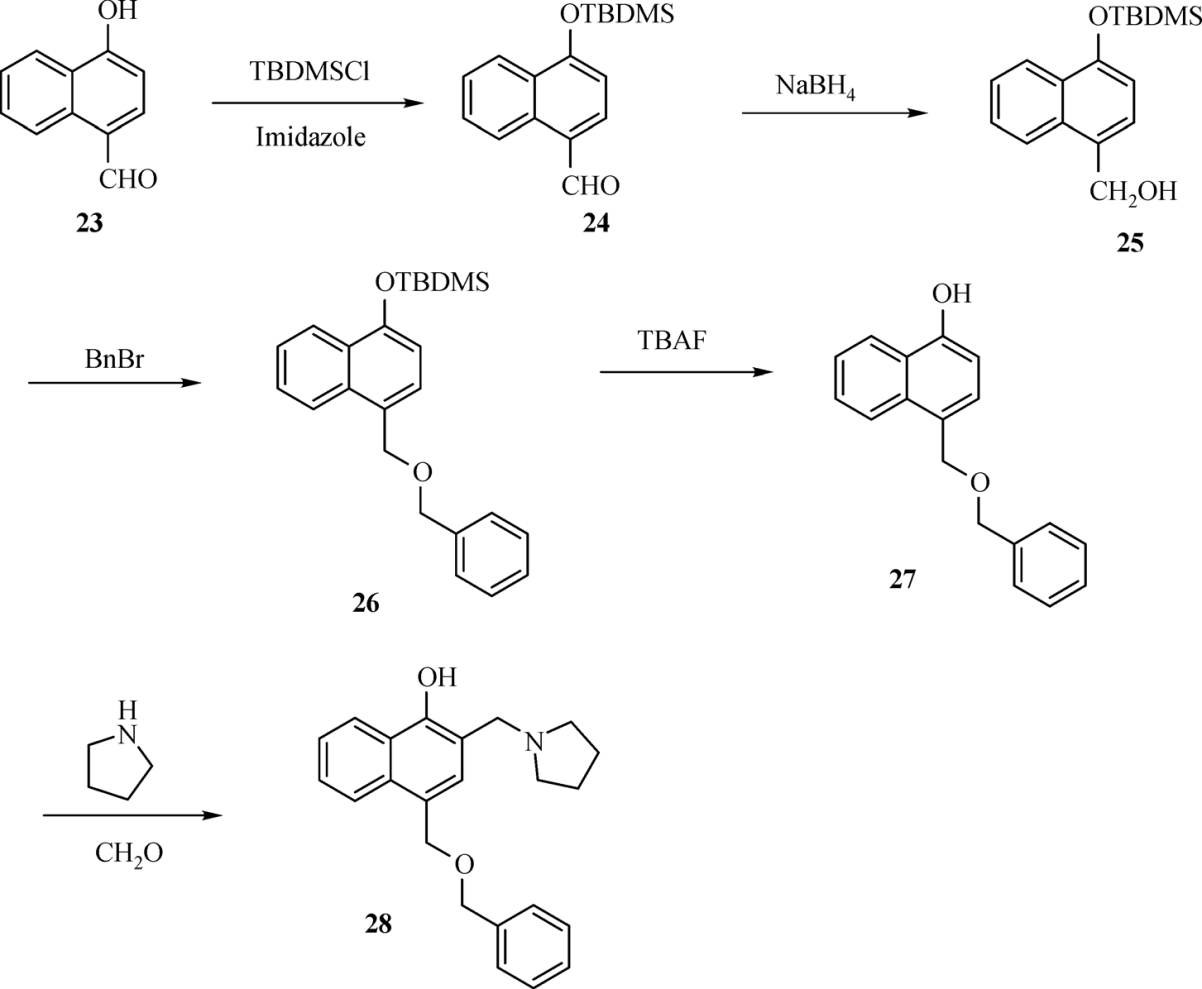
Synthesis of Compound **28**

**Fig.8:**
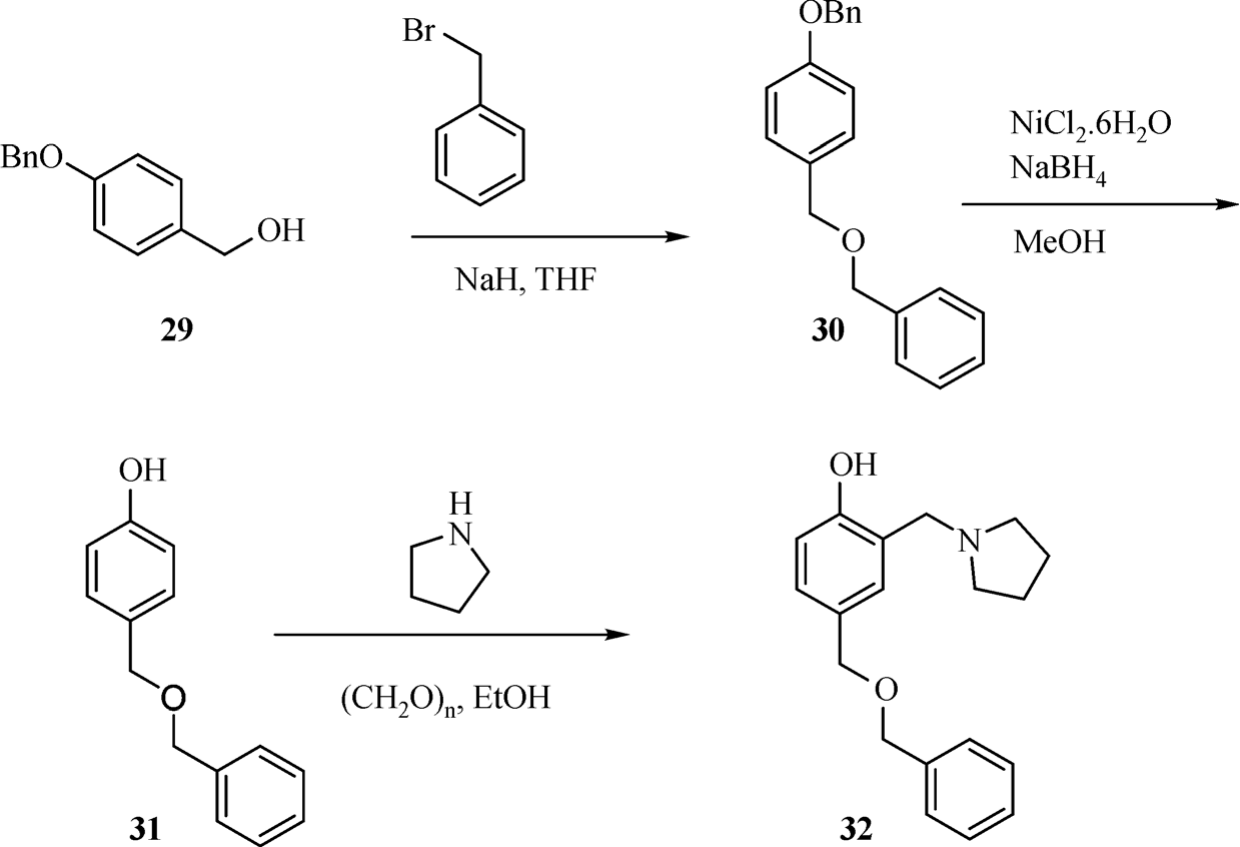
Synthesis of Compound **32**

**Fig.9:**
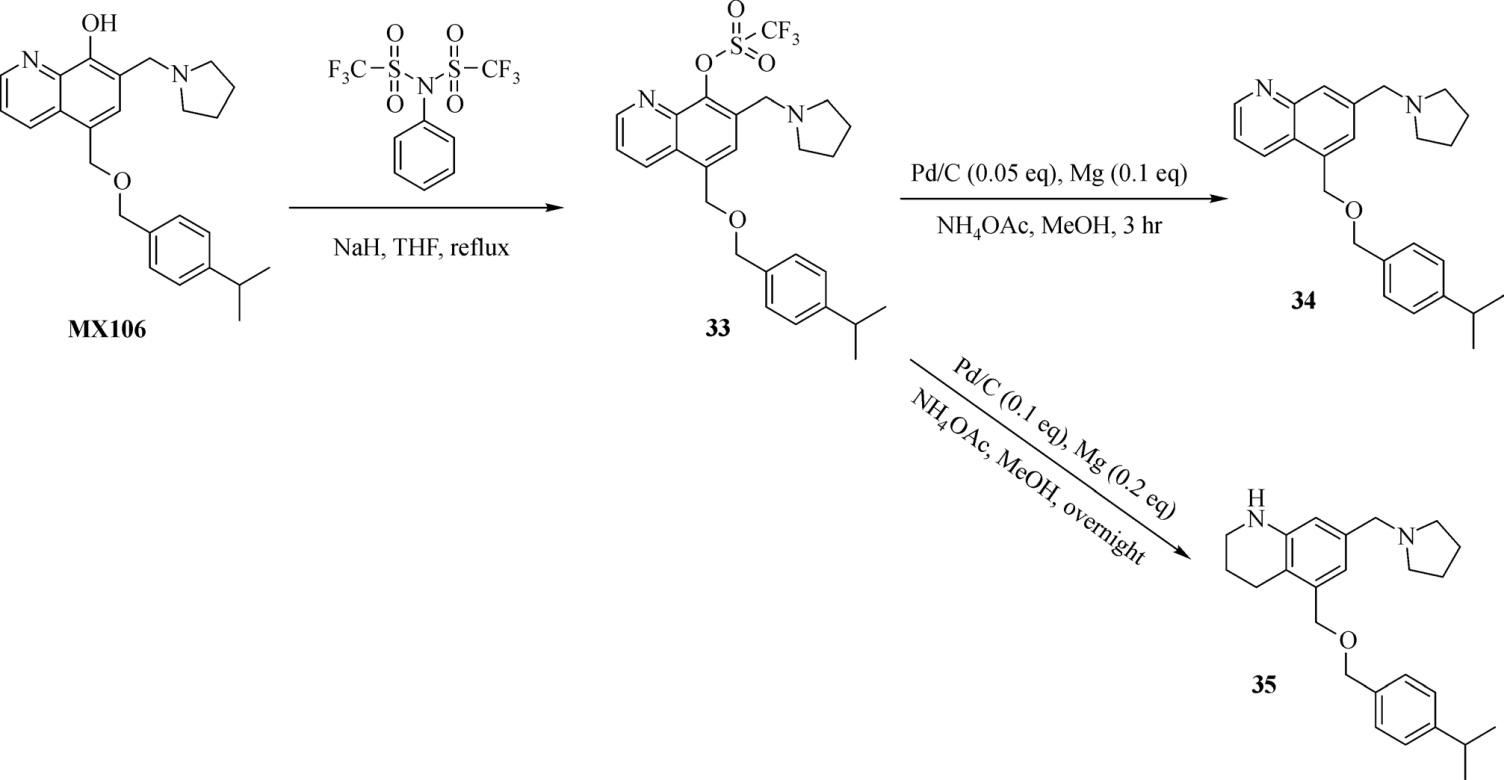
Synthesis of Compounds **33-35**

**Fig.10:**
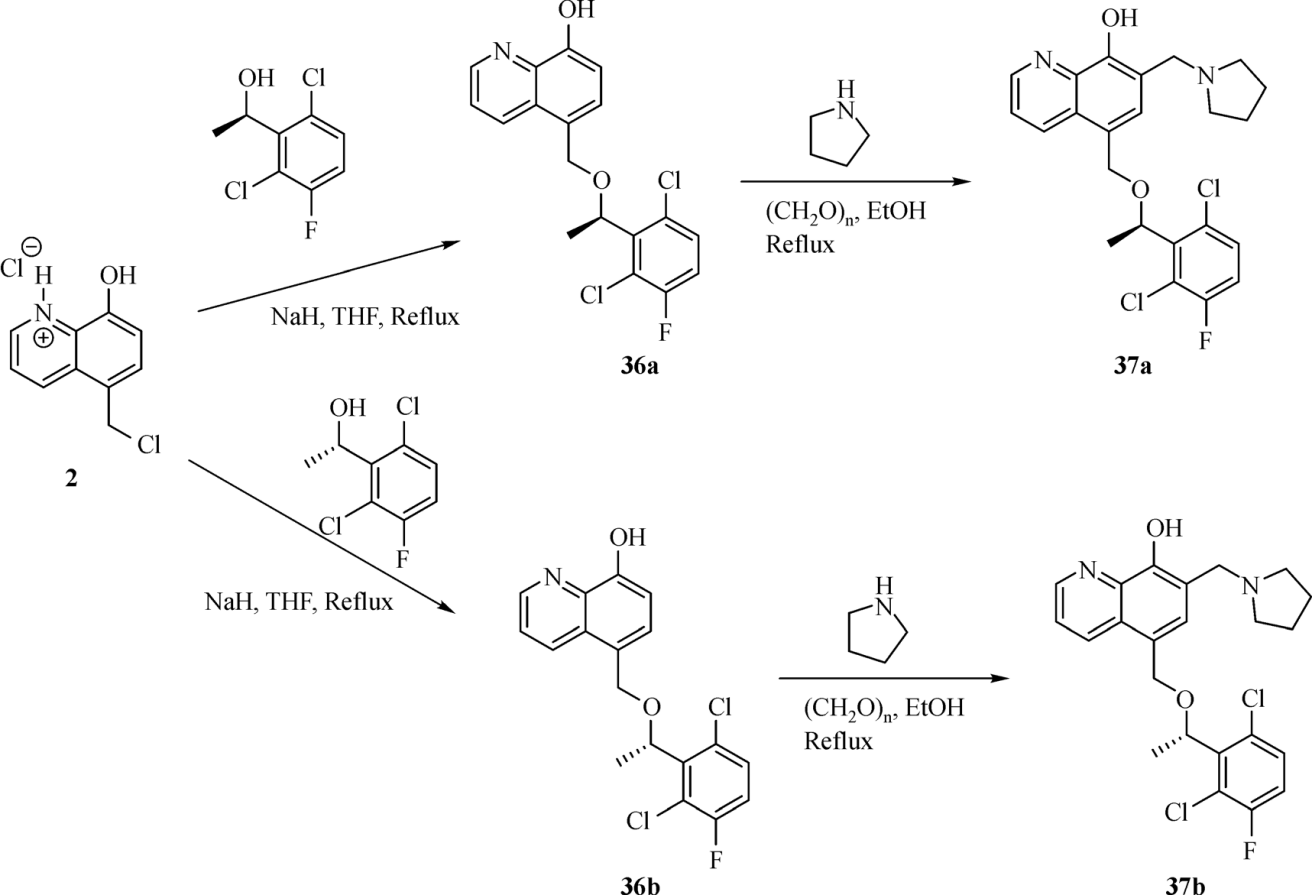
Synthesis of Compounds **37a-b**

**Fig.11:**
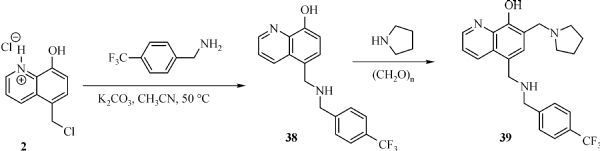
Synthesis of Compound **39**

The general synthesis of C ring substituted **UC112** analogs **4a-4c** is shown in ***Fig. 2***. The procedure is similar to the one reported in our recent findings^[26]^. First, salt **2** was generated, which then reacted with different substituted benzyl alcohols to form ethers **3a-3c**. Then ether **3a-3c** were submitted to Mannich reaction with paraformaldehyde and pyrrolidine to form compounds **4a-4c**. The synthesis of compound **6** applied similar method as compound **4a-4c**, which is shown in ***Fig. 3***. Compound **8** which has morpholine ring on the C ring position was prepared as ***Fig. 4*** shown. First, salt **2** reacted with morpholine to form intermediate **7**. Then intermediate **7** was converted to compound **8** through Mannich reaction. The synthesis of compounds **13-17** is outlined in ***Fig. 5***. Quinoline-2,8-diol first reacted with thionyl chloride in DMF to generate intermediate **10**, which was converted to intermediate **11** by reacting with formaldehyde and catalytic zinc chloride in concentrated hydrochloric acid. Then intermediate **11** was heated in 4-isopropylbenzyl alcohol to form ether **12**. After ether **12** is obtained, it underwent two ways. In the first way, ether **12** went through Mannich reaction to form compound **13**. In the second way, the chloro group on A ring of ether **12** is replaced with 4-methylpiperazine to generate compound **14**. Compounds **15-17** are synthesized from compound **13**. Compound **15** was produced through the reaction of Compound **13** with Isopropyl thiol. Compound **16** was obtained through the reaction of 4-methylpiperazine with Compound **13**. Replacing the pyrrolidine ring on Compound **13** with imidazole ring generated compound **17**. Compound **22**, which has bromo on A ring, was prepared as ***Fig. 6*** shown. The hydroxyl group on quinoline-2,8-diol was protected by acetate group^[32]^. Then, the bromo group was introduced to A ring to form intermediate **19**, which was converted to intermediate **20** by reacting with formaldehyde and catalytic zinc chloride in concentrated hydrochloric acid. Intermediate **20** was heated in heated in 4-isopropylbenzyl alcohol to form ether **21**. Ether **21** then underwent Mannich reaction to produce compound **22**. Compound **28** which replaces the nitrogen on the A ring with Carbon was synthesized as ***Fig. 7*** shown. Aldehyde **23** was first protected by TBDMS group. Then the protected aldehyde **24** was reduced to form alcohol **25**, which reacted with benzyl bromide to form ether **26**. De-protection of ether **26** with TBAF generated intermediate **27**. Then intermediate **27** was converted to compound **28***via* Mannich reaction. Compound **32** which has no A ring was prepared as ***Fig. 8*** shown. Alcohol **29** was first reacted with benzyl bromide to form ether **30**, which was converted to intermediate **31** by removing the benzyl group on phenol position using the method reported in literature^[33]^. Intermediate **31** then underwent Mannich reaction to generate compound **32**. The synthesis of compound **34** and **35** is shown ***Fig. 9***. First triflate group was introduced to MX106 to form compound **33**. Removal of the OTf group produced compound **34**. Reduction of compound **33** with Pd/Carbon generated compound **35**. Compounds **37a-b** were prepared as ***Fig. 10*** shown. First ethers **36a-b** were synthesized. Then they underwent Mannich reaction to form compounds **37a-b**. The amine linked analog, compound **39** was prepared as ***Fig. 11*** shown. Salt **2** first reacted with amine to form intermediate **38**, which was converted to compound **39** through Mannich reaction.

### Biological Results

#### Effect of C ring modification on MX106

The *in vitro* activity for C ring modified MX106 analogs, compounds **4a-4c**, **6** and **8** in A375 and M14 melanoma cells is shown in ***Table 1***. Replacing the isopropyl group with an azide group (**4a**) or an ethynyl group (**4b**) resulted in lower activity than MX106 in both cell lines (1.6 µmol/L and 1.41 µmol/L *vs*. 0.73 µmol/L in A375; 1.4 µmol/L, and 1.38 µmol/L *vs*. 0.72 µmol/L in M14, for **4a**, **4b**, and MX106, respectively). However, introducing two substitution groups (**4c**) to the C-ring or replacing the substituted phenyl ring with a naphthalene ring (**6**) maintain the activity compared with that of MX106 in both A375 cell line (0.75 µmol/L and 0.82 µmol/L *vs*. 0.73 µmol/L, for **4c** and **6,** respectively) and M14 cell (0.75 µmol/L and 0.62 µmol/L *vs*. 0.72 µmol/L). These results suggest that a more lipophilic group at C ring position is slightly favorable for activity. Consistent with this, compound **8** which replaced the phenyl ring with less lipophilic ring morpholine caused significantly decrease of activity (29.09 µmol/L *vs*. 0.73 µmol/L in A375, 16.65 µmol/L *vs*. 0.73 µmol/L in M14). These results further confirmed our previous SAR studies on this ring^[26]^.

**Tab.1:** Effects of structural modifications for MX106 on antiproliferative activity.

**Modifications**	**Compound**		**IC**_**50**_**±SEM (μmol/L)**		**Average**
			**A375**	**M14**	
**C-ring**	**4a**		1.63±0.07	1.40±0.06	1.52
	**4b**		1.41±0.08	1.38±0.04	1.40
	**4c**		0.75±0.03	0.75±0.03	0.75
	**6**		0.82±0.02	0.62±0.01	0.72
	**8**		29.09±0.83	16.65±0.82	22.87
**A-ring**	**13**		7.11±0.44	10.00±10.00	8.55
	**22**		9.11±0.31	9.37±0.32	9.21
	**16**		24.07±0.54	31.39±0.50	27.75
	**15**		9.32±0.49	8.61±0.40	8.95
	**28**		>50	>50	>50
**D-ring**	**17**		7.16±0.21	10.74±0.30	8.95
	**14**		4.64±0.21	9.68±0.35	7.14
**B-ring**	**33**		>50	>50	>50
	**34**		>50	>50	>50
	**35**		12.37±0.45	13.0±0.6	12.69
**Linker**	**37a**		0.65±0.04	0.72±0.05	0.69
	**37b**		0.80±0.05	0.83±0.05	0.82
	**39**		3.44±0.12	3.80±0.15	3.62
	**MX106**		0.73±0.04	0.72±0.05	0.72

#### Effect of A ring modification on MX106

As shown in ***Table 1***, all these modifications resulted in significantly loss of activity. Introducing a chloro (**13**), a bromo (**22**), or an isopropyl thiol group (**15**) to the A ring of MX106 all decreases the anti-proliferative activity compared with that of MX106. The addition of a bulky group as 4-methylpiperazine further decreased activity. Furthermore, replacing the nitrogen atom with a carbon atom on the A ring (**28**) or complete removal of this ring (**32**) caused total loss of activity. These *in vitro* results demonstrate that introduction of substitutions or removal of this pyridine ring of MX106 is not tolerable, and that an unsubstituted pyridine ring on the A ring positon is optimal for activity.

#### Effect of D ring modification on MX106

Some D ring modifications were also performed based on the A ring modified analogs. The *in vitro* activity for the D ring modified analogs, compounds **14** and **17** are also shown in ***Table 1***. Compound **14** which removed D ring did not improve activity compared to MX106. Compound **17** which replaced pyrrolidine ring with imidazole ring was less active than MX106 as well. This result suggests the pyrrolidine ring in this scaffold is needed for activity.

#### Effect of B ring modification on MX106

Since B ring on the UC112 scaffold haven't been modified before, we made several B ring modified analogs, compound **33-35**. As shown in ***Table 1***, Compound **33** which protects the hydroxyl group on the B ring with triflate group was basically inactive, which means the free hydroxyl group is essential for activity. Compound **34**, which removes the hydroxyl group on the B ring, was also inactive. This further confirms the requirement of a free hydroxyl group on B ring for activity. Compound **35** which reduced the pyridine ring to a saturated ring improved activity compared with its unsaturated form **34**, but it is still less active than MX106.

#### Effect of linkers between B ring and C ring of MX106

Finally, we modified the linker between B ring and C ring of MX106. Three compounds **37a**, **37b** and **39** were made as a result. As shown in ***Table 1***. Compound **37a** which introduced a methyl group to the linker was slightly more active than MX106 in A375 (0.65 µmol/L *vs*. 0.73 µmol/L in A375) and has equivalent activity in M14 cell. The added methyl group makes the linker more conformationally rigid, which may explain for the increase of activity. The stereo isomer **37b** was slightly less active than MX106 (0.80 µmol/L *vs*. 0.73 µmol/L in A375, 0.83 µmol/L *vs*. 0.72 µmol/L in M14), which suggests there might be a steric requirement for activity. Replacing the oxygen atom in MX106 with NH (compound **39**) decreases activity (3.44 µmol/L *vs*. 0.73 µmol/L in A375, 3.80 µmol/L *vs*. 0.72 µmol/L in M14).

In general, this systematic modification on different parts of MX106 did not provide significant improvement to its activity, suggesting there are limited structural modifications that can be tolerated in this scaffold to maintain the efficiency for disrupting protein-protein interactions.

#### MDR evaluation results of MX106 analogs

In order to evaluate whether our new compounds can overcome MDR, we selected four most potent compounds, **4c**, **6**, **37a** and **37b** along with MX106, and submitted them to two pairs of cell lines for *in vitro* anti-proliferative activity test. One pair was P-gp overexpressed SW620/Ad300 cell line and its parental, drug-sensitive cell line SW620. The other pair was P-gp overexpressed KB-C2 cell line and its parental, drug-sensitive cell line KB-3-1. The IC_50_ values of the five compounds on the parental cancer cell lines and their P-gp overexpressing resistant sublines were summarized in ***Table 2*** and ***Table 3***. From ***Table 2***, all MX106 analogs showed significantly higher potency in drug resistant cell line SW620/Ad300 than its parental cell line SW620. The five analogs all had very small values of fold resistance. Compound **37a** has a fold resistance as low as 0.07. Similarly, as shown in ***Table 3***, MX106 analogs were more active in drug resistant cell line KB-C2 than its parental cell line KB-3-1. Although existing anticancer drug paclitaxel and survivin inhibitor YM155 were more potent than MX106 analogs in parental sensitive cell lines SW620 and KB-3-1, they essentially lost activities in resistant cell lines SW620/Ad300 and KB-C2. For P-gp substrates YM155 and paclitaxel, strong drug resistance was observed on SW620/Ad300 cell line (5931.42- and 65.25-fold, respectively) and on KB-C2 cell line (7549.38- and 529.42-fold, respectively). On the contrary, cisplatin, which is not a substrate of P-gp, showed similar sensitivity or low resistance (1.8- to 5.1-fold) in drug resistant cells compared to the parental cells. Similarly, the survival curves were made by plotting the survival rate of cell lines at various drug concentrations (***Supplemental Fig. S1*** and ***Supplemental Fig. S2***). As shown in ***Fig. S1*** and ***Fig. S2***, P-gp substrates YM155 and paclitaxel made an obvious shift to right in survival curves of KB-C2 and SW620/Ad300 in comparison with those of the parental cell lines KB-3-1 and SW620, whereas cisplatin exhibited close survival curves between parental and resistant cell lines. In contrast, P-gp overexpressing cell lines treated with our analogs had the survival curves shifting to left compared to their parental cell lines, indicating that the analogs were able to not only overcome the MDR resulting from P-gp overexpression, but also kill the resistant cells at a lower drug concentration.

**Tab.2:** Cytotoxic effects of five analogs on parental SW620 and P-gp overexpressing SW620/Ad300 cell lines.

**Compound**	**IC**_**50**_**±SD**^**a**^** (μmol/L)**		**Fold Resistance**^**b**^
	**SW620**	**SW620/Ad300**	
****MX106	0.15±0.01	0.03±0.01**	0.19
****6	0.13±0.03	0.02±0.01*	0.17
****4c	0.68±0.20	0.07±0.05*	0.10
****37a	0.07±0.02	0.02±0.01*	0.22
****37b	0.14±0.03	0.02±0.01*	0.14
****YM-155	0.0039±0.0007	23.37±2.37**	5931.42
****Paclitaxel	0.030±0.001	1.97±0.36**	65.25
****Cisplatin	1.79±0.06	5.12±0.65*	2.85

^a^IC_50_ is the concentration that inhibited cell survival by half. Data are represented as mean±SD of at least three independent experiments performed in triplicate. ^b^Fold resistance was calculated by dividing the IC_50_ values of resistant cell lines by the IC_50_ values of parental cells. *P<0.05, **P<0.01 *vs.* the values observed in SW620 cell line.

**Tab.3:** Cytotoxic effects of five analogs on parental KB-3-1 and P-gp overexpressing KB-C2 cell lines.

**Compound**	**IC**_**50**_**±SD**^**a**^** (μmol/L)**		**Fold Resistance**^**b**^
	**KB-3-1**	**KB-C2**	
**MX106**	1.25±0.10	0.33±0.12**	0.26
**6**	1.41±0.31	0.21±0.06*	0.15
**4c**	4.33±0.95	0.38±0.06*	0.09
**37a**	1.13±0.11	0.08±0.02**	0.07
**37b**	1.05±0.15	0.15±0.07**	0.14
**YM-155**	0.0049±0.0005	37.30±3.97**	7549.38
**Paclitaxel**	0.0005±0.0001	0.25±0.01**	529.42
**Cisplatin**	1.15±0.13	1.73±0.11*	1.50

^a^IC_50_ is the concentration that inhibited cell survival by half. Data are represented as mean±SD of at least three independent experiments performed in triplicate. ^b^Fold resistance was calculated by dividing the IC_50_ values of resistant cell lines by the IC_50_ values of parental cells. *P<0.05, **P<0.01 *vs.* the values observed in KB-3-1 cell line.

### Mechanism of action studies

Our previous study has established that MX106 produces its antiproliferative activity by selectively inhibiting survivin expression. To determine whether the new analogs maintain their mechanisms of action, we selected two of the most potent MX106 analog, compound **6** and **37a**, and using Western blot analysis to evaluate their inhibitory profile against a panel of IAP proteins.

**Fig.12:**
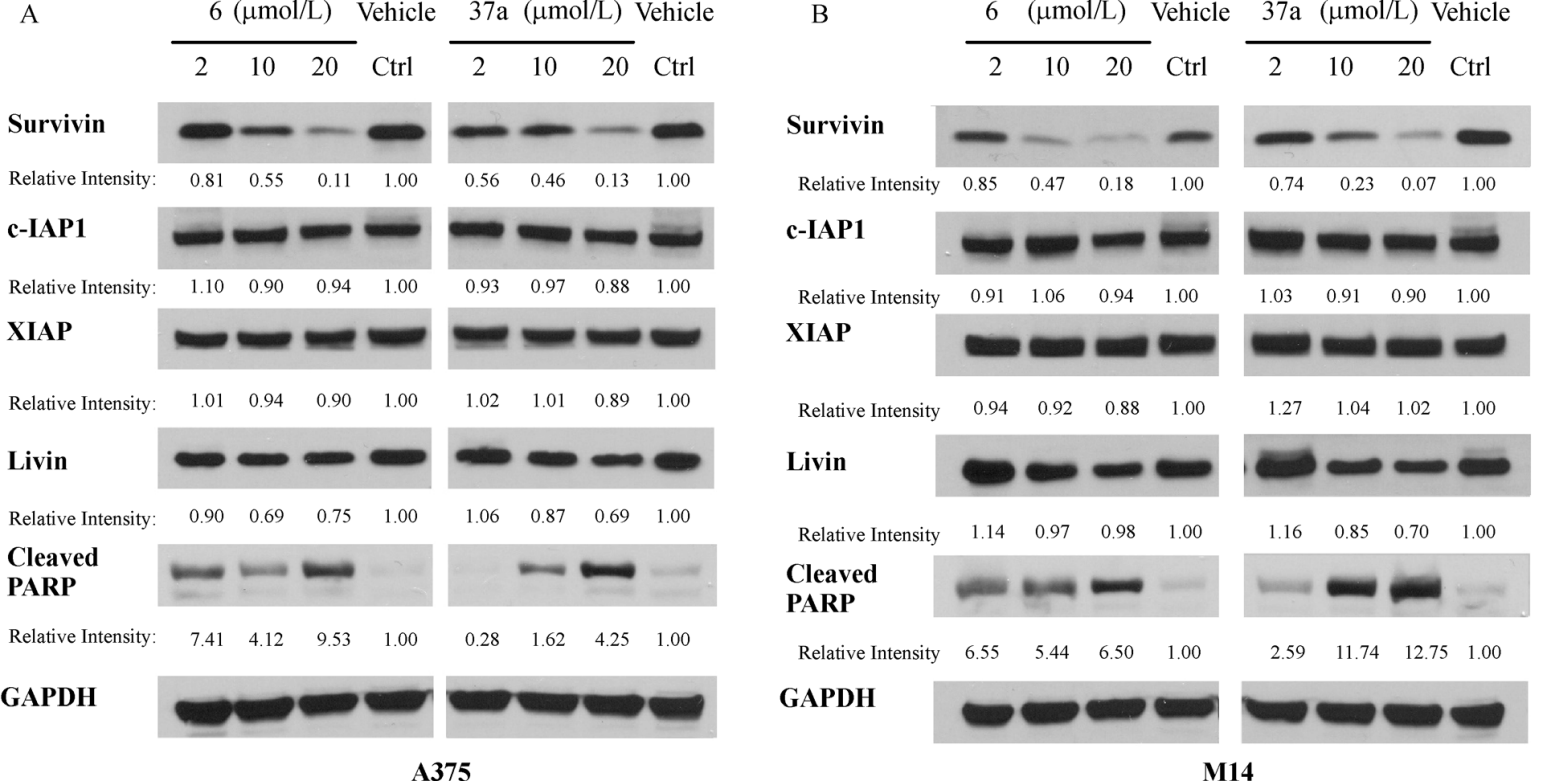
Western blotting of representative compounds **6** and **37a** in suppressing survivin and its closely related IAP proteins in A375 cells (panel A) and M14 cells (panel B).

 As shown in ***Fig. 12***, compound **6** and **37a**, which showed comparatively potent cytotoxicity in tested cancer cell lines, indeed displayed selective and dose-dependent survivin inhibition effects on human melanoma A375 and M14 cells after 24 hours treatment at concentrations from 2 to 20 µmol/L. At the highest concentration tested (20 µmol/L), both compound **6** and **37a** effectively reduced over 80% or 90% survivin level in A375 or M14 cells, respectively. No significant inhibition effect was detected in other IAP proteins including cIAP1, XIAP, and Livin. In addition, we noticed that treatment of compound **6** or **37a** significantly increased the level of cleaved PARP in both cell lines, which is an important apoptosis marker and main cleavage product of the activated caspase-3.

## Discussion

In this work, we performed systematic structural modifications to our previously reported MX106 scaffold. SAR was determined through structural modifications to the A ring, B ring, C ring, D ring and the linker of MX106 scaffold. The SAR information will provide useful directions for future development. The most potent compound **37a** had IC_50_ value in nanomolar range in several cancer cell lines tested in this report. Compound **37a** is more potent than the best compound MX106 reported previously.

The MDR evaluation result suggests that our compounds could effectively overcome P-gp mediated MDR and surprisingly induce more potent cytotoxic effects on P-gp overexpressing cells than their parental cells. A similar finding reported by Ludwig, et al. showed that a thiosemicarbazone derivative NSC73306 was more cytotoxic to isogenic KB cell lines with high P-gp expression than to the P-gp-negative KB-3-1 cell line^[34]^. However, the precise mechanism of the phenomenon remains unclear. Previous studies suggested that survivin plays an important role in MDR in the presence of P-gp, and that inhibition of survivin could sensitize MDR cells to anticancer drugs^[35^-^37]^. It was reported that in the KB cell line, human breast cancer MCF-7 cell line, and their corresponding P-gp overexpressing cell lines KBv_200_ and MCF-7/ADR, neither upregulation nor downregulation of survivin could affect P-gp expression on protein level^[34]^, but the PI3k/Akt pathway was found to be involved in P-gp associated survivin transcription activity and affected the expression of both P-gp and survivin in the same pattern in MCF-7/ADR^[36]^. These suggested that survivin in MDR cancer cells may interact with P-gp indirectly. Although these findings might be cell-type specific, we could not eliminate the possibility that the compounds in our study may achieve circumvention of P-gp-mediated MDR through a similar mechanism indirectly affecting P-gp and survivin. It is also possible that by similar mechanism of action on P-gp, our new analogs could overcome MDR mediated by other ABC transporters.

We are currently working on to determine the activity of MX106 against other clinically relevant drug resistance mechanisms and will report our findings in the future. Mechanistically, Western blotting analyses confirmed that the new compounds maintained the same mode of action as MX106 by selectively suppressing survivin expression levels. The excellent activity of those new survivin inhibitors warrants further development.
